# Clinical and Microbiological Profile of Oral Candidiasis: A Retrospective Study

**DOI:** 10.3390/pathogens15020195

**Published:** 2026-02-10

**Authors:** Maja Ptasiewicz, Karolina Thum-Tyzo, Alicja Matejko, Julia Georges, Emanuela Bis, Aleksandra Strączek, Renata Chałas, Agnieszka Magryś

**Affiliations:** 1 Department of Oral Medicine, Medical University of Lublin, 20-093 Lublin, Poland; karolina.thum-tyzo@umlub.edu.pl (K.T.-T.); renata.chalas@umlub.edu.pl (R.C.); 2 Student Research Group at the Department of Oral Medicine, Medical University of Lublin, 20-093 Lublin, Poland; 3 Doctoral School, Medical University of Lublin, 20-093 Lublin, Poland; 4 Department of Medical Microbiology, Medical University of Lublin, 20-093 Lublin, Poland; agnieszka.magrys@umlub.edu.pl

**Keywords:** oral candidiasis, denture stomatitis, *Candida albicans*

## Abstract

**Introduction**: Oral candidiasis is a common opportunistic fungal infection of the oral mucosa, most frequently caused by *Candida albicans*. Its development is influenced by local factors, such as denture use and oral hygiene, as well as systemic conditions including diabetes, nutritional deficiencies, and chronic inflammatory diseases. Accurate diagnosis requires both clinical evaluation and mycological testing. The aim of this retrospective study was to analyze demographic characteristics, predisposing factors, and the species distribution of Candida isolates in patients diagnosed with oral candidiasis. **Materials and Methods**: A retrospective review of medical documentation was conducted to evaluate patient demographics, risk factors, comorbidities, denture use, and results of mycological examinations confirming oral candidiasis. **Results**: A total of 71 patients (49 women and 22 men), aged 21–85 years (mean 59.6 ± 16 years), were included in the study. Fungal etiology was confirmed in all cases, with *Candida albicans* identified most frequently (81.69%). Among comorbidities, cardiovascular diseases were most common (30.99%), followed by diabetes (14.08%), and chronic periodontitis, respiratory, and gastrointestinal diseases (each 11.27%). Removable dentures were used by 18.30% of patients, and nicotine addiction was reported in 9.86%. All strains were susceptible to the tested antifungals, except for species with known intrinsic resistance. **Conclusions**: Oral candidiasis in this cohort predominantly affected women and older adults, with *Candida albicans* remaining the most common etiological agent. Denture use emerged as an important local predisposing factor and was associated with a higher proportion of infections caused by non-albicans species. These findings underscore the importance of comprehensive clinical evaluation and routine mycological testing to guide targeted antifungal therapy, especially in patients with risk factors such as denture use or systemic comorbidities.

## 1. Introduction

Oral candidiasis is a common fungal infection affecting the mucous membranes of the oral cavity. Its etiology is multifactorial, primarily associated with infections caused by Candida species, most frequently *Candida albicans*, which is isolated from approximately 80% of lesions [1]. Other species such as *Candida glabrata*, *C. tropicalis*, *C. krusei*, *C. guillermondii*, *C. lusitaniae*, *C. parapsilosis*, *C. pseudotropicalis*, and *C. stellatoidea* may also be responsible for infection [2]. Local predisposing factors include poor denture hygiene, poorly fitting dentures, and xerostomia, which create favorable conditions for fungal colonization. Systemic factors, such as iron and folic acid deficiencies, diabetes mellitus, and immunosuppression, also significantly contribute to the development of oral candidiasis [3]. Oral candidiasis presents in several clinical forms recognized in medical practice, including acute pseudomembranous candidiasis, acute erythematous (atrophic) candidiasis, post-antibiotic candidiasis, and chronic forms such as chronic atrophic candidiasis (denture stomatitis), angular cheilitis, median rhomboid glossitis, and chronic hyperplastic candidiasis (candidal leukoplakia) [1,4]. Each form arises in response to distinct predisposing factors. Numerous variables influence susceptibility to oral candidiasis, including age, gender, and the presence of dental prosthetics. Understanding these risk factors and their role in the pathogenesis of oral candidiasis is essential for effective treatment and prevention. Age is a significant factor influencing susceptibility. Older individuals often have weakened immune systems, which promote fungal infections such as candidiasis [1,5]. Additionally, reduced salivary flow, common in elderly patients, limits natural mechanical and enzymatic protection against *Candida* spp., increasing their vulnerability to infection [6]. Gender may also play a role in disease occurrence. Research suggests that women are more frequently affected than men [5], likely due to hormonal influences on the oral microbiota. Factors such as pregnancy, hormone replacement therapy, and oral contraceptive use may further increase susceptibility. The presence of dental prosthetics, particularly dentures, is a well-established risk factor for oral candidiasis. Materials used in denture fabrication may support Candida colonization [7]. Additionally, prosthetic appliances can cause mucosal irritation and compromise local defense mechanisms, facilitating fungal overgrowth [1,8]. Denture stomatitis typically manifests as erythema and swelling of the oral mucosa in direct contact with the prosthesis. Symptoms may include burning, pain, or may be entirely asymptomatic. Lesions can appear in various areas of the oral cavity, including the tongue, buccal mucosa, palate, lips, gingiva, and posterior pharyngeal wall. They are usually asymmetrical, presenting as white plaques that can be easily removed, often revealing erythematous or bleeding mucosa underneath [1]. OC presents in one of two forms: white or erythematous. White OC is characterized by lesions that are white, including pseudomembranous candidiasis, hyperplastic candidiasis chronic mucocutaneous candidiasis. Erythematous OC is characterized by lesions that are red, including chronic atrophic candidiasis-denture stomatitis, median rhomboid glossitis, angular cheilitis, antibiotic or steroids-inducted stomatitis and erythematous candidiasis in HIV patients [1,8].

Acute erythematous candidiasis is characterized by diffuse redness and inflammation, often accompanied by burning, pain, and, in some cases, difficulty chewing or swallowing [3]. Diagnosis and treatment of different clinical forms of oral candidiasis require thorough clinical evaluation and an individualized approach, tailored to the underlying causes and symptomatology [9].

The infection is typically treated with antifungal drugs, primarily azoles, polyenes, and synthetic echinocandins. While echinocandins act directly on the fungal cell wall, polyenes and azoles—in addition to disrupting the cell membrane by interfering with the synthesis and distribution of ergosterol—can also affect cholesterol synthesis in the host’s eukaryotic cells. Therefore, they exhibit potential hepatotoxic and nephrotoxic effects. Side effects and drug resistance are also possible, especially with long-term or repeated use of these drugs [10]. The effectiveness of any antifungal therapy depends primarily on correcting the local and systemic factors that promote fungal infections. Uncorrected, recurrent oral candidiasis can lead to increasingly severe symptoms and an increased tendency for microbial dissemination, which in half of cases can be associated with serious consequences, including the development of candidemia and dissemination of infection from the oral cavity into the bloodstream. An innovative approach to eliminating fungal infections involves the use of probiotics, which are based on their ability to restore the balance of a healthy microbiota [11].

The aim of this study was to retrospectively evaluate the association between selected risk factors—such as denture use, smoking, and comorbidities—and the occurrence of oral candidiasis, and to characterize the distribution of Candida species among affected patients. The main research question was whether certain demographic or clinical factors are statistically associated with specific patterns of Candida species identified in patients diagnosed with oral candidiasis. Clarifying these relationships may improve clinical risk assessment and inform prevention strategies.

## 2. Materials and Methods

### 2.1. Study Design and Data Collection

A retrospective analysis of electronic patient medical records was conducted to evaluate the relationship between selected risk factors and the distribution of *Candida* species in individuals diagnosed with oral candidiasis. The study included data from 71 patients (22 males and 49 females) who attended the University Dental Clinic in Lublin, Poland between January 2022 and January 2023 for consultation and treatment due to suspected oral candidiasis.

Inclusion criteria comprised: (1) clinical suspicion of oral candidiasis and (2) the availability of complete medical documentation, including microbiological test results. Exclusion criteria were: (1) incomplete patient records, (2) lack of confirmation of candidiasis by mycological examination, and (3) age under 18 years.

Data were extracted retrospectively from each patient’s medical file, focusing on demographic characteristics (age, gender), medical history, and known predisposing factors such as denture use, tobacco smoking, and concomitant systemic diseases. The results of mycological examinations performed to confirm the diagnosis were also reviewed.

### 2.2. Clinical and Mycological Evaluation

The clinical diagnosis of oral candidiasis was based on the patient’s history and intraoral examination, following the classification proposed by Holmstrup et al. [12], distinguishing pseudomembranous, erythematous, and hyperplastic forms, as well as other *Candida*-related lesions (prosthetic stomatitis, angular cheilitis, median rhomboid glossitis, linear gingival erythema).

Biological samples for mycological analysis were collected in the form of swabs from the oral mucosa and/or the surface of dentures. Samples were processed by a certified external laboratory in collaboration with our department.

Cultures were performed on Sabouraud Dextrose Agar (Argenta, Poznań, Poland). Yeast isolates were identified using the VITEK^®^ 2 Compact automated system with YST identification cards (bioMérieux, Craponne, France), following the manufacturer’s instructions. Briefly, pure yeast colonies grown on Sabouraud Dextrose Agar were suspended in sterile saline to obtain a standardized inoculum (0.5–2.0 McFarland). The suspension was inoculated into YST cards containing a panel of biochemical assays based on carbohydrate assimilation and metabolic activity. Cards were incubated and analyzed automatically by the VITEK^®^ 2 system. Species identification was established by comparison of the generated biochemical profiles with the system’s reference database, and only identifications meeting the manufacturer-defined confidence criteria were included in the analysis.

Antifungal susceptibility data were extracted retrospectively from laboratory records. Susceptibility of *Candida* isolates had been assessed in the diagnostic laboratory as part of routine clinical testing, performed in accordance with the standards and internal procedures in place at the time of analysis. The antifungal panel included ketoconazole, nystatin, fluconazole, itraconazole, miconazole, econazole and clotrimazole. Since EUCAST or CLSI breakpoints are not available for several of these agents, results were interpreted according to the laboratory’s internal interpretive criteria and known intrinsic resistance patterns reported in the literature (e.g., intrinsic fluconazole resistance in *C. krusei* and reduced fluconazole susceptibility in *C. glabrata*) [13].

### 2.3. Assessment of Risk Factors

For each patient, local and systemic risk factors potentially predisposing to *Candida* infection were analyzed. Local factors included the use of removable dentures (duration of use, whether worn continuously or removed overnight), tobacco use and oral hygiene status.

Systemic conditions with potential immunosuppressive effects were also considered, including diabetes mellitus, hypothyroidism, neurological disorders, cardiovascular diseases, respiratory diseases, skin disorders, gastrointestinal disorders, rheumatologic diseases, hematologic disorders, urinary system disorders, neoplastic diseases. Additionally, tobacco smoking was considered both as a local and systemic risk factor due to its potential impact on oral mucosal immunity and overall health.

### 2.4. Data Management and Statistical Analysis

All extracted data were entered into Microsoft Excel for organization and preliminary evaluation. Statistical analyses were performed using Statistica software (version 7.1, StatSoft, Tulsa, OK, USA). Descriptive statistics (frequencies, percentages, means, and standard deviations, where applicable) were used to characterize demographic variables, *Candida* species distribution, and the prevalence of individual risk factors.

Categorical variables, including the distribution of *Candida* species in relation to local and systemic risk factors, were compared using Fisher’s exact test due to the small sample size in several subgroups. A *p*-value < 0.05 was considered statistically significant.

### 2.5. Ethical Statement

This study was conducted as a retrospective observational analysis based on routinely collected clinical documentation. All data were fully anonymized prior to analysis, and no patient-identifiable information was accessed. All patients had previously provided standard written informed consent for diagnostic and therapeutic procedures, including mycological examinations, as part of routine clinical care. The retrospective use of anonymized data for scientific analysis did not require additional informed consent or approval by a bioethics committee, in accordance with national regulations and institutional policies.

## 3. Results

### 3.1. Oral Candidiasis and the Age of Patients

**Figure 1:**
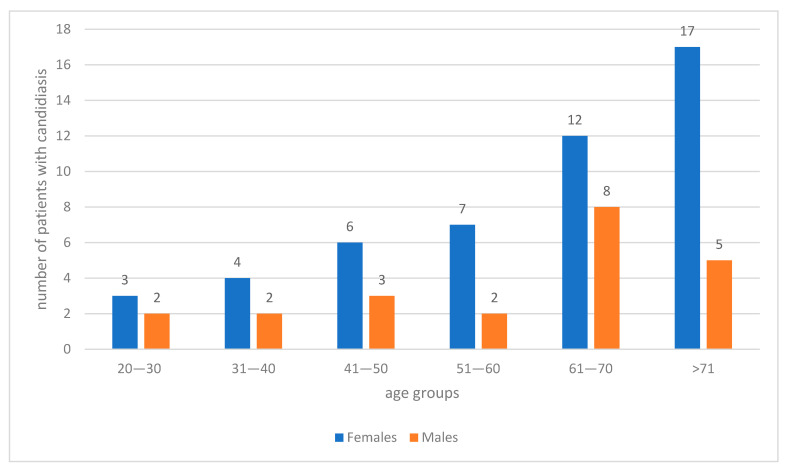
Distribution of patients with oral candidiasis by age group and gender.

The study group included 71 patients, comprising 49 women (69.0%) and 22 men (31.0%), aged 21–85 years, with a mean age of 59.6 ± 16.0 years. Fungal infection was confirmed in all patients (100%) and occurred significantly more often in women than in men (49/71, 69.0% vs. 22/71, 31.0%; *p* = 0.019). The highest number of cases (22 patients, 31.0%) was observed in the age group over 70 years, while the lowest number of cases (5 patients, 7.0%) occurred in the youngest age group (20–30 years) (Figure 1). Of note, among female patients of reproductive (20–50 years, *n* = 13) or menopausal age (>50 years), none were pregnant, none reported the use of oral contraceptives, and none reported using hormone replacement therapy at the time of diagnosis.

### 3.2. Candida Species Among Patients

**Figure 2:**
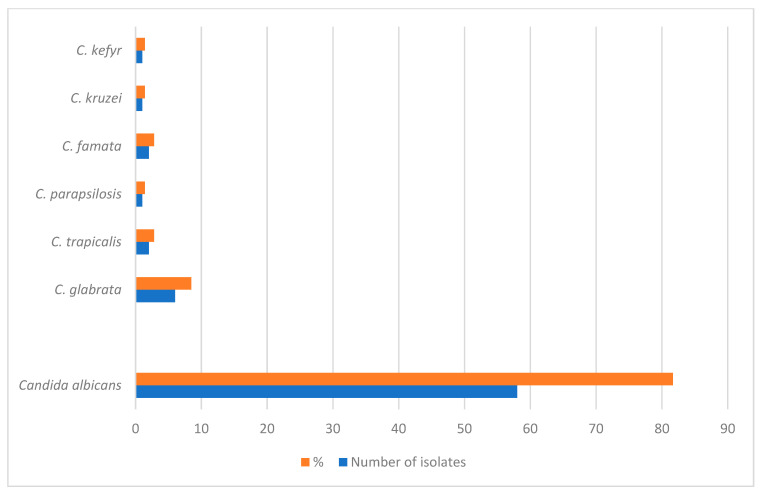
Distribution of Candida species among patients with oral candidiasis.

*Candida albicans* was the predominant species, identified in 81.69% of patients. Other species included *Candida glabrata* (8.45%), *Candida tropicalis* (2.81%), and *Candida famata* (2.81%). Less frequently observed species were *Candida kefyr*, *Candida krusei*, and *Candida parapsilosis*, each accounting for 1.41% of cases (Figure 2).

### 3.3. Type and Location of Oral Candidiasis Among Patients

Among the 71 patients, in 20 (28.17%) acute pseudomembranous candidiasis (“thrush”) localized on the tongue, buccal mucosa, hard and soft palate were diagnosed, 11 (15.49%) suffered from chronic hyperplastic candidiasis of the commissures of the mouth. 19 (26.76%) of the examined patients had angular cheilitis (angular stomatitis or perlèche) localized in the commissures of the mouth. 7 patients had median rhomboid glossitis (9.86%) on the dorsal tongue, and 14 (19.71%) patients were diagnosed with denture stomatitis (chronic atrophic candidiasis) of hard palate and mandibular mucosa (Table 1).

### 3.4. Systemic Diseases Among Patients

Systemic diseases were present in 44 patients (61.9%). Cardiovascular diseases were the most common (30.99%), followed by diabetes (14.08%), respiratory diseases, and gastrointestinal disorders (each 11.27%). Hypothyroidism was diagnosed in 8.45% of patients, while urinary system diseases were observed in 7.04%. All neoplastic conditions (*n* = 5; 7.04%) identified in the study were benign (2 uterine fibroids and 3 lipomas). No malignant neoplasms, including oral cancer, were recorded. Dermatological, neurological, hematological, and rheumatological conditions were less frequent. No statistically significant relationship was found between *Candida* species and the presence of comorbidities (*p* = 0.344) (Table 2).

### 3.5. Local Risk Factors and Their Association with Candida Species

Local risk factors were identified in a subset of patients and showed varying associations with *Candida* species. Chronic periodontal disease was observed in 8 patients (11.27%), and in all cases *C. albicans* was the only species isolated. The association between periodontal disease and *Candida* species distribution was not statistically significant (*p* = 0.336).

Nicotine addiction was reported in 7 patients (9.86%). Among them, *C. albicans* was identified in 71.4% and non-*albicans* species in 28.6% of cases. No statistically significant association was found between smoking status and the isolated species (*p* = 0.604).

Denture use was documented in 13 patients (18.30%)—10 patients had partial dentures (14.1%) and 3 had total dentures (4.2%). In this group, non-albicans species were more common (53.8%) than *C. albicans* (46.2%). A statistically significant relationship was observed between denture use and the presence of non-albicans species (*p* = 0.001) (Table 3).

### 3.6. Antifungal Susceptibility of Isolated Candida Species

The susceptibility of the isolated *Candida* species was tested using ketoconazole, nystatin, fluconazole, itraconazole, miconazole, econazole, and clotrimazole. All strains were susceptible to the tested antifungals, except for species with known intrinsic resistance.

*Candida albicans* remained fully susceptible to all tested drugs, including fluconazole. Among non-albicans species, most isolates were susceptible, but *C. krusei* is naturally resistant to fluconazole, and *C. glabrata* shows reduced susceptibility or dose-dependent susceptibility (Table 4).

## 4. Discussion

In this retrospective study, oral candidiasis was diagnosed in 71 patients, predominantly female (69.01%). This gender distribution is consistent with epidemiological reports suggesting that women are more susceptible to oral *Candida* spp. colonization, potentially due to hormonal modulation of the oral microbiota and immune response [13,14,15,16,17]. Although no statistically significant association between gender and *Candida* species was observed in our cohort, the clear predominance of females aligns with global trends, emphasizing the importance of considering sex-specific factors in oral fungal infections.

The mean age of participants was 59.6 ± 16 years, reflecting the well-established correlation between increasing age and susceptibility to *Candida* colonization. Advanced age is associated with immunosuppression, reduced salivary flow, and alterations in mucosal integrity, all of which facilitate fungal overgrowth [16]. Our data also suggest a trend toward increased colonization by non-albicans species in patients over 70 years, particularly *C. glabrata*, which is consistent with prior studies indicating that aging favors colonization by species with higher adherence and antifungal tolerance [17,18]. Although not statistically significant, this observation highlights a potential clinical consideration for elderly populations, where non-albicans *Candida* may complicate treatment strategies [19].

Local factors, specifically denture use, emerged as a significant determinant of species distribution. *Candida* non-albicans species were more frequent in denture users (53.85% vs. 10.53%, *p* = 0.001), suggesting that prosthetic appliances may alter oral microenvironments to favor colonization by less common *Candida* species. These findings corroborate previous work demonstrating that denture surfaces, particularly when worn long-term or poorly maintained, promote adhesion and biofilm formation by *C. glabrata*, *C. tropicalis*, and *C. krusei* [19,20,21]. The clinical implications are noteworthy: both denture hygiene and the choice of prosthetic materials may influence not only the overall risk of developing oral candidiasis but also the composition of colonizing *Candida* species, which in turn can shape antifungal treatment strategies.

Systemic comorbidities were present in 61.97% of patients, with cardiovascular diseases and diabetes being the most common. While our analysis did not reveal significant associations between *Candida* species and comorbidities (*p* = 0.344), these underlying conditions remain important contributors to oral fungal susceptibility. For example, hyperglycemia in diabetes creates a nutrient-rich environment in saliva, enhancing *Candida* growth and biofilm formation [22,23,24,25]. Cardiovascular diseases may further influence susceptibility through microvascular changes and immune dysregulation [26,27,28,29,30]. Thus, even in the absence of statistically significant associations, the biological plausibility supports their role as risk modifiers.

Cigarette smoking was reported in 9.86% of patients and was not significantly associated with *Candida* species. While low prevalence in this cohort limits statistical power, smoking is known to impair mucosal immunity and promote adhesion and biofilm formation by *C. albicans* [31,32,33]. The lack of significant association may reflect sample size limitations rather than absence of effect, reinforcing the need for larger studies or pooled analyses.

Antifungal susceptibility testing confirmed that *C. albicans* remained fully susceptible to all tested agents, including fluconazole. Non-*albicans* species generally responded to treatment, with the exception of intrinsic fluconazole resistance in *C. krusei* and dose-dependent reduced susceptibility in *C. glabrata*. These results are consistent with EUCAST guidelines and prior literature [34,35,36], underscoring the clinical relevance of accurate species identification for guiding therapy. Even in a relatively small cohort, the detection of *Candida* non-albicans species with known resistance profiles highlights the importance of targeted mycological diagnostics in clinical practice [37].

Taken together, our findings reinforce the multifactorial nature of oral candidiasis. Age, denture use, systemic conditions, and microbial species characteristics collectively shape susceptibility and treatment response. While statistical significance was limited in some analyses, the observed trends are consistent with established biological mechanisms and suggest actionable insights for clinical practice.

## 5. Conclusions

Oral candidiasis in this cohort predominantly affected women and older individuals, with *C. albicans* being the most prevalent species. Denture use was associated with higher rates of non-albicans colonization, emphasizing the importance of prosthesis hygiene and monitoring. Although systemic diseases and smoking were not statistically associated with species distribution, their known biological impact on oral fungal colonization warrants consideration in patient management. Fluconazole demonstrated robust activity against *C. albicans*, but non-albicans species such as *C. glabrata* and *C. krusei* exhibited intrinsic or dose-dependent resistance, highlighting the need for species-specific antifungal selection.

These results underscore the importance of comprehensive oral care, targeted mycological diagnostics, and individualized therapeutic strategies, particularly for older patients, denture users, and those with systemic comorbidities. However, several limitations of this study should be acknowledged. The relatively small sample size may have reduced the statistical power to detect significant associations between oral candidiasis, smoking status, and systemic comorbidities. Despite these limitations, the findings provide clinically relevant insights into the distribution of *Candida* species and associated risk factors in patients with oral candidiasis and highlight important directions for future prospective, multicenter studies with larger cohorts aimed at further clarifying the relationships between risk factors, *Candida* species distribution, and antifungal susceptibility.

## Figures and Tables

**Table 1:** Type and location of oral candidiasis in patients.

Form of Candidiasis	Number of Patients	Location
Acute pseudomembranous candidiasis (“thrush”)	20 (28.17%)	tongue, buccal mucosa, hard palate, soft palate
Chronic hyperplastic candidiasis	11 (15.49%)	commissures of the mouth
Angular cheilitis (angular stomatitis or perlèche)	19 (26.76%)	commissures of the mouth
Median rhomboid glossitis	7 (9.86%)	dorsal tongue
Denture stomatitis (chronic atrophic candidiasis)	14 (19.71%)	hard palate, mandibular mucosa

**Table 2:** Prevalence of systemic diseases among patients with oral candidiasis.

Systemic Predisposing Factor	Total No. of Cases (%)	Females	Males
Cardiovascular diseases	22 (30.99%)	13	9
Diabetes mellitus	10 (14.08%)	4	6
Hypothyroidism	6 (8.45%)	5	1
Neurological disorders	3 (4.22%)	2	1
Respiratory diseases	8 (11.27%)	3	5
Skin disorders	4 (5.6%)	3	1
Gastrointestinal disorders	8 (11.27%)	4	4
Rheumatologic diseases	1 (1.4%)	1	0
Hematologic disorders	1 (1.4%)	0	1
Urinary tract disorders	5 (7.04%)	1	4
Benign neoplastic diseases	5 (7.04%)	3	2

**Table 3:** Local risk factors and their association with *Candida* species.

Local Risk Factor	No. of Cases (%)	Association with *Candida albicans* Species (%)	Association with Non-Albicans Species (%)
Chronic periodontal disease	8 (11.27%)	8 (100%)	0 (0%)
Nicotine addition	7 (9.86%)	5 (71.4%)	2 (28.5%)
Denture use (total) - partial dentures - total dentures	13 (18.3%) 10 (14.1%) 3 (4.2%)	6 (46.15%) 4 (40%) 2 (66.6%)	7 (53.8%) 6 (60%) 1 (33.3%)

**Table 4:** Antifungal susceptibility of isolated *Candida* species.

Species	KTZ	FLU	ITC	NYS	MIZ	ECO	CLO
*C. albicans* (*n* = 58)	S	S	S	S	S	S	S
*C. glabrata* (*n* = 6)	S	I	S	S	S	S	S
*C. tropicalis* (*n* = 2)	S	S	S	S	S	S	S
*C. famata* (*n* = 2)	S	S	S	S	S	S	S
*C. parapsilosis* (*n* = 1)	S	S	S	S	S	S	S
*C. kefyr* (*n* = 1)	S	S	S	S	S	S	S
*C. krusei* (*n* = 1)	S	R *	S	S	S	S	S

KTZ—Ketoconazole; FLU—Fluconazole; ITC—Itraconazole; NYS—Nystatin; MIZ—Miconazole; ECO—Econazole; CLO—Clotrimazole; S—susceptible; I—susceptible, increased exposure; R—resistant (intrinsic); * *C. krusei* is intrinsically resistant to fluconazole.

## Data Availability

The data presented in this study are not publicly available due to ethical and legal restrictions related to patient confidentiality. Anonymized data may be made available from the corresponding author upon reasonable request and with appropriate approvals.
